# Cultural differences in ant-dipping tool length between neighbouring chimpanzee communities at Kalinzu, Uganda

**DOI:** 10.1038/srep12456

**Published:** 2015-07-22

**Authors:** Kathelijne Koops, Caspar Schöning, Mina Isaji, Chie Hashimoto

**Affiliations:** 1 Anthropological Institute and Museum, University of Zurich, Winterthurerstrasse 190, 8057 Zürich, Switzerland; 2 Department of Archaeology and Anthropology, University of Cambridge, Pembroke Street, CB2 3QG Cambridge, United Kingdom; 3 Institut für Biologie, Arbeitsgruppe Funktionelle Biodiversität, Freie Universität, Königin-Luise-Strasse 1-3, 14195 Berlin, Germany; 4 Primate Research Institute, Kyoto University, Aichi 484-8506, Inuyama, Japan

## Abstract

Cultural variation has been identified in a growing number of animal species ranging from primates to cetaceans. The principal method used to establish the presence of culture in wild populations is the method of exclusion. This method is problematic, since it cannot rule out the influence of genetics and ecology in geographically distant populations. A new approach to the study of culture compares neighbouring groups belonging to the same population. We applied this new approach by comparing ant-dipping tool length between two neighbouring communities of chimpanzees (*Pan troglodytes schweinfurthii*) in the Kalinzu Forest, Uganda. Ant-dipping tool length varies across chimpanzee study sites in relation to army ant species (*Dorylus* spp.) and dipping location (nest vs. trail). We compared the availability of army ant species and dipping tool length between the two communities. M-group tools were significantly longer than S-group tools, despite identical army ant target species availabilities. Moreover, tool length in S-group was shorter than at all other sites where chimpanzees prey on epigaeic ants at nests. Considering the lack of ecological differences between the two communities, the tool length difference appears to be cultural. Our findings highlight how cultural knowledge can generate small-scale cultural diversification in neighbouring chimpanzee communities.

Cultural phenomena have been identified in a growing number of animal species, ranging from primates to cetaceans^1^,^2^,^3^. The principal method used to establish culture in wild animal populations is the method of exclusion^1^. This method identifies geographically variable behaviour patterns across study sites and seeks to exclude those variants that can be attributed to genetic or ecological differences across sites. Problematically, this method cannot conclusively exclude the influence of genetic and environmental factors^4^,^5^,^6^, especially since comparisons generally involve geographically distant populations. Moreover, cultural processes interact with genetics^7^ and ecology^8^, in terms of innate predispositions and ecological opportunities for tool use.

Therefore, a novel approach to the study of animal material culture was developed aimed at minimizing the influence of genetics and ecology: comparing neighbouring groups belonging to the same population^9^. Studying genetically similar groups living under very similar environmental conditions allows for investigation of fine scale cultural differences, whilst keeping genetics constant. However, (subtle) ecological differences between neighbouring communities still need to be excluded. The argument for cultural differences between neighbouring groups is especially convincing when immigrating individuals can be observed to change their behaviour according to the customs of their new group^10^.

Recent studies have examined in detail the variation between neighbouring chimpanzee communities in the Taï Forest, Ivory Coast^9^,^10^,^11^. Community differences in a foraging context were found in the selection of hammers for nut-cracking^9^,^10^, as well as in termite prey selection and in strategies of army ant nest raiding^11^. These differences were interpreted to be cultural, since nut-cracking tool material availability, termite mound abundance and army ant prey characteristics could not explain the community differences. Moreover, a new immigrant female at Taï was observed to behave progressively more similar to her new group with regards to tool selection for nut-cracking^10^, thus further supporting a cultural transmission process. A cultural interpretation would be strengthened if such differences between neighbouring communities were to be replicated in other populations. Here, we report a between-community difference in the length of tools employed in harvesting army ants by chimpanzees in East Africa.

Dipping for army ants (*Dorylus* spp.) is one of the hallmark examples of culture in chimpanzees^12^,^13^. In ant-dipping, chimpanzees use a stiff wand of woody or herbaceous vegetation to extract the highly aggressive army ants from their temporary underground nests^14^ or directly from surface trails^15^,^16^. Army ants are ubiquitous across chimpanzees study sites, and chimpanzees use tools to prey on them at over a dozen sites^17^.

Previous research has shown that dipping tool length is functionally linked to army ant prey type, dipping location and technique^15^,^17^,^18^. First, longer tools are used for more aggressive army ant species (‘epigaeic’), and shorter tools for less aggressive species (‘intermediate’). This pattern was found both within community^15^ and across populations^17^. Second, chimpanzees at Bossou (Guinea) use longer tools for the same army ant type when dipping at nests as compared to trails^17^. Third, tool length is linked to ant-dipping technique. Two main techniques exist. In the ‘pull-through’ method, a chimpanzee dips a tool into a nest (or trail) of army ants, waits for the ants to swarm up the tool, then withdraws it using a hand or foot, sweeps of the ants with the other hand and transfers the ants to the mouth^14^. In the ‘direct-mouthing’ method, a chimpanzee dips for ants and then directly sweeps the tool through the mouth or nibbles the ants off the tool^19^. The ‘pull-through’ technique is associated with longer tools (>50 cm) and the ‘direct-mouthing’ technique with shorter tools^15^.

In this study, we investigated differences in ant-dipping tool length between two neighbouring communities of chimpanzees (*Pan troglodytes schweinfurthii*) in the Kalinzu Forest, Uganda. Kalinzu is a suitable site to investigate cultural differences in ant-dipping tool use between adjacent communities. The two study groups at Kalinzu, M-group and S-group, share the same continuous forest block and have partly overlapping home ranges with regular female transfers between the two groups. Hence, if we find differences in tool length between neighbouring communities *without* finding differences in army ant species availability, these differences are highly likely to have a cultural basis.

We measured availability of army ants along transects covering both chimpanzee communities’ ranges. In addition, we recorded recently used ant-dipping sites and tools during daily follows of members of both communities. In the fully habituated M-group, we also directly observed ant-dipping and recorded tool characteristics, dipping techniques and army ant species targeted. We examined the relationship between the availability of the different army ant species and their consumption by M-group chimpanzees. Moreover, we analysed the relationship between army ant species targeted and dipping tool length.

## Results

### Ant-dipping sites

**Figure 1:**
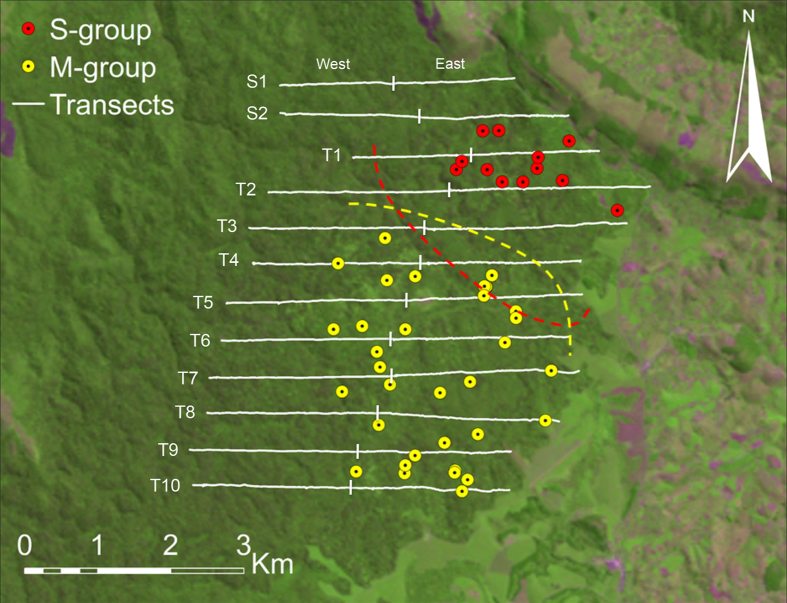
Ant-dipping sites in the two neighbouring communities at Kalinzu. Ant-dipping sites in M-group (yellow) and S-group (red) with transect lines (white) and approximate home range boundaries for both groups (dashed). Map created using ESRI ArcMap 10.1 and Landsat image.

We recorded 35 ant-dipping sites with 145 tools in M-group and 12 ant-dipping sites with 41 tools in S-group (Fig. 1). All ant-dipping sites recorded were at army ant nests. The mean number of tools per site was 4.1 tools (median = 4.0, SD = 2.0, *n* = 35, range: 1–9) for M-group and 3.3 tools (median = 2.5, SD = 2.5, *n* = 12, range: 1–9) for S-group, which was a non-significant difference (Mann-Whitney U test: z = −1.471, *P* = 0.141).

### Tool characteristics

**Figure 2:**
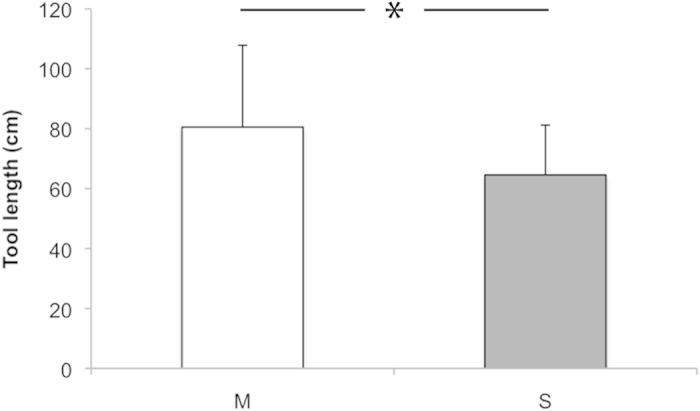
Ant-dipping tool length in the two neighbouring communities at Kalinzu. Mean ant-dipping tool length in cm (SD) for M-group (white) and S-group (grey). *MWU test: P < 0.0001.

**Figure 3:**
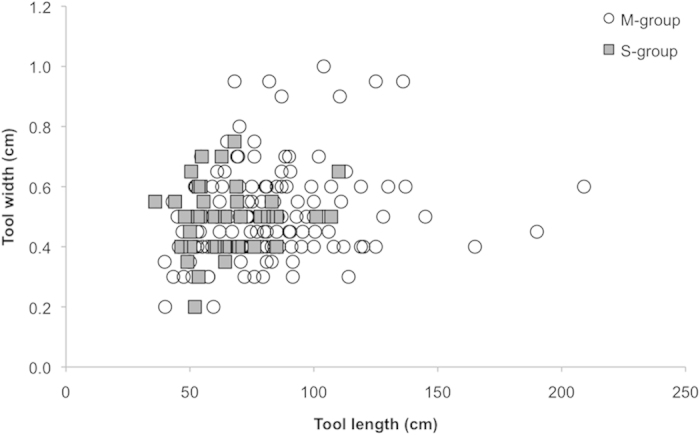
Ant-dipping tool dimensions in the two neighbouring communities at Kalinzu. Tool length and tool width for M-group (white dots) and S-group (grey squares).

Mean tool length for M-group was 80.5 cm (median = 76.0, SD = 27.3, *n* = 145, range: 39.8–209.0 cm) versus 64.6 cm (median = 62.8, SD = 16.6, *n* = 41, range: 36.0–110.0 cm) for S-group. Tools used by M-group were significantly longer than tools used by S-group (Mann-Whitney U test: z = −3.848, *P* < 0.0001, Fig. 2 & Fig. 3).

Mean tool width for M-group tools was 0.50 cm (median = 0.50, SD = 0.15, *n* = 145, range: 0.20–1.00) and for S-group mean tool width was 0.49 cm (median = 0.50, SD = 0.11, *n* = 41, range: 0.20–0.75). Tool width did not differ significantly between the two groups (Mann-Whitney U test: z = −0.100, *P* = 0.921, Fig. 3).

### Ant availability

First, we compared ant trail densities between M-group and S-group ranges. The mean ant trail density for M-group was 0.55 trails/km (SD = 0.32, *n* = 17, range: 0–1.4). The mean ant trail density for S-group was 0.42 trails/km (SD = 0.23, *n* = 13, range: 0 – 0.80). Army ant trail densities did not differ between the two groups (Independent Samples T-test: t = −1.29, df = 28, *P* = 0.206).

Second, we compared the availability of army ant species between M-group and S-group ranges. In M-group, 93.0% of samples were *D. terrificus* and 4.7% were *D. wilverthi* (total: *n* = 43). *D. terrificus* and *D. wilverthi* are epigaeic species. In addition, one sample (2.3%) in M-group belonged to an intermediate species (*D. kohli*), not eaten by the chimpanzees. In S-group, 95.0% of samples were *D. terrificus* and 5.0% were *D. wilverthi* (total: *n* = 20). The proportions of *D. terrificus* and *D. wilverthi* samples did not differ between the two groups (Fisher’s Exact test: *P* = 1.00). Hence, army ant target species availability was the same for M- and S-group.

### Ant species availability and consumption

In M-group, we compared the availability of *D. terrificus* and *D. wilverthi* with the consumption of these two species*. D. terrificus* made up 93.0% of *all occurrences* collected ant samples (*n* = 187) and 92.3% of dipping sites for which ant species could be identified (*n* = 26). Hence, the M-group chimpanzees consumed the two species according to their availability (Fisher’s Exact test: *P* = 1.000).

### Ant species and tool characteristics

We compared the length of tools used in M-group to prey on *D. terrificus* vs. *D. wilverthi*. Mean tool length for *D. terrificus* was 79.0 cm (median = 76.0, SD = 25.6, *n* = 91, range: 40.0–190.0 cm). Mean tool length for *D. wilverthi* was 104.5 cm (median = 101.3, SD = 38.6, *n* = 6, range: 63.0–165.0 cm). Tool length did not differ significantly between the two species (Mann-Whitney U test: z = 1.72, *P* = 0.085). Mean tool width for *D. terrificus* was 0.50 cm (median = 0.50, SD = 0.15, *n* = 91, range: 0.2–1.0 cm). Mean tool width for *D. wilverthi* was 0.46 cm (median = 0.45, SD = 0.11, *n* = 6, range: 0.3 – 0.6 cm). Tool width also did not differ between the two species (Mann-Whitney U test: z = −0.645, *P* = 0.519).

## Discussion

Ant-dipping tool length differed between the two neighbouring chimpanzee communities at Kalinzu, despite them having identical army ant availabilities. The question therefore is: *Why* do M-group chimpanzees use longer ant-dipping tools than S-group chimpanzees?.

The availability of the two epigaeic army ant species, *D. terrificus* and *D. wilverthi*, was the same in both communities. Thus, prey species availability could not explain the tool length difference. Moreover, chimpanzees in M-group preyed on the two army ant species according to their availability. Similarly, unhabituated chimpanzees at Seringbara, Guinea, and Gashaka, Nigeria, consume army ant species present according to encounter probabilities^20^,^21^. S-group chimpanzees most likely also consumed army ant species opportunistically and according to their availability. Hence, the use of different length tools for the same prey species suggests a cultural difference between the two communities.

**Figure 4:**
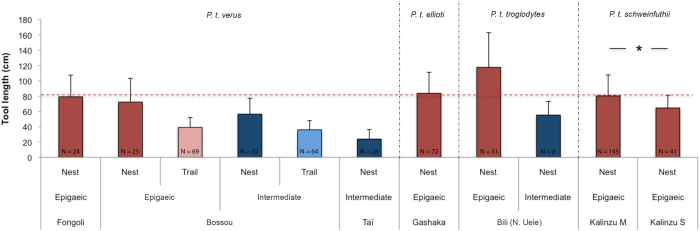
Ant-dipping tool length across chimpanzee study sites. Mean tool length in cm (SD) across chimpanzee study sites according to army ant type (epigaeic vs. intermediate) and dipping location (nest vs. trail). Red dashed line indicates the mean tool length for epigaeic nests. N indicates the number of dipping tools for each site. *MWU test: P < 0.0001. References for each site: Fongoli^24^, Bossou^17^, Taï^19^, Gashaka^22^,^23^, Bili (N. Uele)^36^, Kalinzu M (This study), Kalinzu S (This study).

The tool length in M-group (80.5 cm) closely resembles tool lengths found at other sites where chimpanzees dip for epigaeic army ants at nests (Fig. 4): 83.8 cm at Gashaka^22^,^23^, 79.3 cm at Fongoli in Senegal^24^ and 72.3 cm (epigaeic nests only) at Bossou in Guinea (Fig. 4). Tool lengths at epigaeic nests did not differ across these sites^17^. However, tool length in S-group was significantly shorter than in M-group, and well below the mean tool length (83.0 cm) at epigaeic nests across sites (Fig. 4). In fact, S-group tool length (64.6 cm) was more similar to sites where chimpanzees prey on both epigaeic and intermediate species: 64.2 cm at Seringbara^20^, 66 cm at Gombe in Tanzania^14^ and 53.7 cm at Bossou^15^. So the real question is: Why does tool length in S-group diverge from other chimpanzee communities preying exclusively at nests of epigaeic army ants?.

One possible explanation for the shorter tools in S-group is that the chimpanzees in this community may use a different dipping technique, requiring shorter tools. At Bossou, chimpanzees generally use the pull-through technique to prey on epigaeic army ant species and the direct-mouthing technique for intermediate species^15^,^17^. At Taï, chimpanzees prey only on intermediate species and use the direct-mouthing technique^19^. At Fongoli, chimpanzees prey only on epigaeic army ants and use the pull-through technique most of the time (J. Pruetz, pers. comm.). If S-group chimpanzees use the direct-mouthing technique, whereas M-group chimpanzees do not, this could explain the difference in tool length.

The M-group chimpanzees were observed to exclusively use the pull-through technique (Table 1). We obtained direct observations for five individuals (3 adult males, 2 adult females) at five dipping sites, all *D. terrificus* nests. Before we can conclude that the direct-mouthing technique is absent in M-group, we first need observations of ant-dipping with short tools (<50 cm). We observed an interesting new ant-dipping technique in M-group, which we termed the ‘staggered pull-through’ technique (Supplementary Video S1). In the ‘staggered pull-through’ method, a chimpanzee withdraws the tool by changing the grip of the tool a number of times whilst feeding the tool through the hand or foot (‘staggering’). This technique was observed with long tools of 100 cm or more (Table 1). The use of the foot to pull through, whilst hanging suspended from one arm, was observed in three of the five individuals, including whilst using the ‘staggered pull-through’ technique (Supplementary Video S2).

The long tools of M-group may also be linked to the use of tree perches from which to dip (Supplementary Videos S1 & S2). Chimpanzees make use of one or more tree saplings to make an elevated perch from which to dip more securely for biting army ants on the ground below^14^. In M-group, 79% (23/29) of dipping sites had a tree perch. This is much higher compared to 40% at Seringbara, where chimpanzees dip for both epigaeic and intermediate army ants^20^. Preliminary observations suggest that S-group chimpanzees regularly dip without using a tree perch (M. Isaji, personal observation). Future research is needed to examine tree perch use in S-group in more detail and to investigate whether or not shorter tools are indeed linked to less frequent use of tree perches in this community. Moreover, physical characteristics of army ant nests (i.e. depth, presence of roots) and prey behaviour (i.e. aggressiveness, speed) in the two study groups may possibly also affect tool length and remain to be addressed in future studies.

The data for S-group are relatively limited and the difference in tool length could potentially reflect a different prey preference in this community. Also, data collection did not cover a full annual cycle and more extensive sampling could improve army ant availability estimates. We were able to identify the target ant species for three dipping sites in S-group, all of which were *D. terrificus*, the most common species. However, even if S-group differs in army ant species preference, this would still mean that there is a cultural difference between the two communities, because prey availabilities are identical. Moreover, both army ant species in Kalinzu are epigaeic and thus are aggressive with long legs and mandibles^17^. This might explain why M-group chimpanzees did not adjust their tool length according to whether they dipped for *D. terrificus* or *D. wilverthi*. Hence, even if S-group chimpanzees preferred one of the two epigaiec army ant species, this still would not provide an explanation for the difference in tool length. Moreover, a cultural interpretation is strengthened by recent observations of S-group chimpanzees using a tool set in ant-dipping, which has never been observed in M-group (Hashimoto *et al*. under review).

The presence of tool length ‘sub-cultures’ in neighbouring communities at Kalinzu raises the question as to how these local cultural differences within a population become established and are maintained. In chimpanzees, most females leave their natal group around the time of sexual maturity to migrate to a new community. At Taï, a female chimpanzee immigrant was observed to adopt the nut-cracking behaviour (i.e. hammer selection) of her new community^10^. Such conformist tendencies in immigrant females can lead to persistent group-typical behaviours. Conformity has also been found to play a role in maintaining group dependent cultural traits in wild vervet monkeys^25^. In the Taï chimpanzees, group specific tool selection remained similar over 25 years^10^. In Kalinzu’s M-group, tool length seems to be stable over time, based on the tool length (79 cm) reported for 1997–1998^26^, which closely resembles tool length found in the current study (80.5 cm). We need long-term data from both study groups to establish whether or not the observed difference in tool length is consistent over time.

In conclusion, ant-dipping tool length in two neighbouring communities in Kalinzu differed significantly, despite identical availabilities of army ant species. Considering the genetic exchange between the two groups and the absence of ecological explanations, the tool length difference was found to be cultural. Conformist tendencies of immigrant females likely support such group typical behaviours. It remains to be investigated in future studies to what extent the tool length difference at Kalinzu is linked to dipping technique.

## Methods

### Study site and subjects

Kalinzu Forest Reserve is in western Uganda 30° 07’ E, 0° 17’ S^27^. The forest is classed as medium-altitude moist evergreen forest^28^,^29^. The main study community (M-group) consisted of 97 individuals (19 adult males, 29 adult females). The neighbouring community (S-group) consisted of 30 individuals (6 adult males, 8 adult females). Data collection took place from December 2012 - March 2013, covering the late rainy season (until end of December) and dry season (early January–mid March)^30^.

### Data collection

KK collected data in M-group and MI collected data in S-group with help of local field assistants. We followed the chimpanzees from approximately 07.30 h until 16.00 h for six days per week. Behavioural data collection involved focal animal sampling and instantaneous scan sampling for party composition and activity^31^. In M-group, ant-dipping tool use was recorded opportunistically whenever a party member was seen to engage in ant-dipping behaviour. In M-group, we filmed the tool use session whenever visibility was sufficient, using a Canon Powershot SX 40 HS. S-group chimpanzees were not fully habituated to human observers and were often difficult to follow on the ground. Hence, ant-dipping behaviour was rarely directly observed in S-group.

In both M-group and S-group, we recorded all recent ant-dipping sites (max. 2 weeks old) encountered in the forest and we collected and measured all tools present. We sampled army ant workers from ant-dipping sites for species identification whenever ants were still present upon finding the exploited nest. Army ant colonies usually emigrate to new nest sites after a predator attack^32^,^33^, thus rendering it difficult to find ants for species identification at dipping sites of more than a few days old. For all ant-dipping tools we recorded the following variables: 1. *Length*: measured with meter tape (in cm); 2. *Width* at midpoint: measured with a caliper (in cm).

Army ant trail densities were recorded along 12 established parallel transects (Fig. 1), each approx. 5 km long and 500 m apart, covering both the home ranges of M-group (38.3 km) and S-group (25.4 km), as well as the overlap area (9.6 km) of the two communities (total transect length: 54 km). We walked transects in February and March 2013. For each army ant column or swarm encountered, we recorded the GPS location with a Garmin Map 60csx. Estimating army ant colony densities from ant trails is an established and reliable method^21^. We sampled workers from each ant column and swarm raid for species identification. To avoid counting the same army ant colony twice, we considered only foraging columns and swarm raids of the same *Dorylus* species to be independent (i.e. belonging to different colonies), when the distance between them was >100 m. If trails or swarm raids of the same species were found within 100 m distance, we counted them only once since colonies may use several trails simultaneously^34^. We estimated army ant species availability in more detail within the M-group’s range by sampling workers from *all* army ant swarms, raids and nests encountered (*n* = 187) during working days in the forest (duration: 59 days, distance walked: 489 km).

### Statistical analyses

We tested data for normality using a normal probability plot and a Kolmogorov-Smirnov test^35^. Non-parametric tests were used when data were not normally distributed. All analyses were two-tailed and significance levels were set at 0.05. We performed statistical tests in IBM SPSS version 21.0. We used Mann-Whitney U tests to compare mean number of tools per dipping site, mean tool length and mean tool width between the two groups.

We calculated the mean army ant density (trails/km) for each transect based on the two surveys in February and March 2013. Each transect consisted of two parts (East and West), both approx. 2.5 km in length (Fig. 1). We used the 2.5 km-sections as the unit of analyses. We used an Independent Samples T-test to compare mean army ant density between the two groups. We used a Fisher’s Exact test to compare army ant species availability between the two groups.

To compare the availability and consumption of army ant species in M-group, we used the ant availability estimate based on all occurrences sampling and the consumption estimate based on dipping sites with ant species identification. We used a Fisher’s Exact test to compare army ant species availability and consumption in M-group. Lastly, we used a Mann-Whitney U test to compare the length of tools used to prey on *D. terrificus* vs. *D. wilverthi* by M-group chimpanzees.

## Additional Information

**How to cite this article**: Koops, K. *et al.* Cultural differences in ant-dipping tool length between neighbouring chimpanzee communities at Kalinzu, Uganda. *Sci. Rep.* **5**, 12456; doi: 10.1038/srep12456 (2015).

## Supplementary Material

Supplementary Information

Supplementary Video S1

Supplementary Video S2

## Figures and Tables

**Table 1:** Information on observed and filmed ant-dipping sessions at *D. terrificus* nests by identified chimpanzees (date, name, sex, technique, hand use, foot use, tool length).

Date	Name	Sex	Technique	Hand	Foot	Tool length (cm)
17.01.13	Gai	F	Pull-through*	L	R	190, 104
17.01.13	Jo	M	Pull-through	–	R	89
30.01.13	Gure	M	Pull-through	R	–	97
28.02.13	Jo	M	Pull-through*	–	L	106
28.02.13	Prince	M	Pull-through	–	L	69
02.03.13	Yosuko	F	Pull-through*	L	–	100
04.03.13	Jo	M	Pull-through*	R	–	130

^*^Staggered pull-through.
