# Supplementary material for: Cultural differences in ant-dipping tool length between neighbouring chimpanzee communities at Kalinzu, Uganda
Source: Sci Rep. 2015 Jul 22;5:12456. doi: 10.1038/srep12456 (PMC4510480; doi:10.1038/srep12456)
Supplement: Supplementary Information [file srep12456-s1.pdf]

Cultural differences in ant-dipping tool length between neighbouring chimpanzee communities at Kalinzu, Uganda

Kathelijne Koops[a,b*], Caspar Schöning[c], Mina Isaji[d] and Chie Hashimoto[d]

[a]*Anthropological Institute and Museum, University of Zurich, Winterthurerstrasse 190, 8057 Zürich, Switzerland*

[b]*Department of Archaeology and Anthropology, University of Cambridge, Pembroke Street, CB2 3QG Cambridge, United Kingdom*

[c]*Institut für Biologie, Arbeitsgruppe Funktionelle Biodiversität, Freie Universität, Königin-Luise-Strasse 1-3, 14195 Berlin, Germany*

[d]*Primate Research Institute, Kyoto University, Aichi 484-8506, Inuyama, Japan*

***Corresponding Author:**

Kathelijne Koops

Anthropological Institute & Museum

University of Zurich

Winterthurerstrasse 190

8057 Zürich

Switzerland

Email: kathelijne.koops@uzh.ch

Supplementary information

Supplementary Video S1. Adult female chimpanzee at Kalinzu (Yosuko) ant-dipping using the ‘staggered pull-through’ technique (© K. Koops).

Supplementary Video S2. Adult female chimpanzee at Kalinzu (Gai) ant-dipping using her foot for the ‘staggered pull-through’ technique (© K. Koops).
